# Application of Novel Amino-Functionalized NZVI@SiO_2_ Nanoparticles to Enhance Anaerobic Granular Sludge Removal of 2,4,6-Trichlorophenol

**DOI:** 10.1155/2015/548961

**Published:** 2015-04-28

**Authors:** Zeyu Guan, Jinquan Wan, Yongwen Ma, Yan Wang, Yajie Shu

**Affiliations:** ^1^College of Environmental Science and Engineering, South China University of Technology, Guangzhou 510006, China; ^2^State Key Laboratory of Pulp and Paper Engineering, South China University of Technology, Guangzhou 510006, China

## Abstract

A novel amino-functionalized silica-coated nanoscale zerovalent iron (NZVI@SiO_2_-NH_2_) was successfully synthesized by using one-step liquid-phase method with the surface functionalization of nanoscale zerovalent iron (NZVI) to enhance degradation of chlorinated organic contaminants from anaerobic microbial system. NZVI@SiO_2_-NH_2_ nanoparticles were synthesized under optimal conditions with the uniform core-shell structure (80–100 nm), high loading of amino functionality (~0.9 wt%), and relatively large specific surface area (126.3 m^2^/g). The result demonstrated that well-dispersed NZVI@SiO_2_-NH_2_ nanoparticle with nFe^0^-core and amino-functional silicon shell can effectively remove 2,4,6-trichlorophenol (2,4,6-TCP) in the neutral condition, much higher than that of NZVI. Besides, the surface-modified nanoparticles (NZVI@SiO_2_-NH_2_) in anaerobic granule sludge system also showed a positive effect to promote anaerobic biodechlorination system. More than 94.6% of 2,4,6-TCP was removed from the combined NZVI@SiO_2_-NH_2_-anaerobic granular sludge system during the anaerobic dechlorination processes. Moreover, adding the appropriate concentration of NZVI@SiO_2_-NH_2_ in anaerobic granular sludge treatment system can decrease the toxicity of 2,4,6-TCP to anaerobic microorganisms and improved the cumulative amount of methane production and electron transport system activity. The results from this study clearly demonstrated that the NZVI@SiO_2_-NH_2_/anaerobic granular sludge system could become an effective and promising technology for the removal of chlorophenols in industrial wastewater.

## 1. Introduction

Chlorophenols (CPs), a group of toxic and suspected carcinogenic pollutants, have been widely identified in chemical industrial wastewater. CPs are considered resistant to biodegradation and may cause adverse effects on human health and the receiving environment [1–3]. The development of technologies for the remediation of CPs from industrial wastewater has attracted a great deal of attention in recent years, such as activated carbon absorption, photocatalytic degradation, electrochemical oxidation, and biodegradation [4–7]. Among these methods, anaerobic biological treatment technology is widely applied in toxic industrial wastewater treatment process due to convenient usage and economy [8, 9]. However, anaerobic microorganisms for treating industrial wastewater containing high concentration of CPs have technical limitations such as low degradation rates, long cleanup times, and inefficient activity of biological system, due to the unfavorable environmental conditions, properties of CPs, and their sterilized effects on microorganisms [10, 11]. Therefore, it is necessary to develop the advanced anaerobic technologies with high biodegradation rates and suitable microbial system for removal and mineralization of CPs into harmless end products from industrial wastewater.

During the last decade, nanoscale zerovalent iron (NZVI) has been widely applied as a promising tool for the treatment of contaminated groundwater and soil [12]. NZVI can remove many kinds of recalcitrant pollutants such as chlorinated organic compounds, azo dyes, nitroaromatic pollutants, and heavy metals through reductive reaction mechanism [13–16]. Besides, NZVI-microorganisms system has also been conducted to investigate the codegradation pattern of chlorinated organics since they maintain a high removal rate and sufficient anaerobic microbial activity [17–19]. The role of NZVI is expected to help create an enhanced microbial environment combining all advantages of physical absorption, chemical reaction, and biodegradation. During the NZVI corrosion/hydrogen reduction reaction, the produced hydrogen gas has been considered as an electron-donor source for anaerobic microorganisms, such as methanogenic, homoacetogenic, sulfate-reducing bacteria and denitrifying bacteria [20–22]. Moreover, the removal of perchlorate, trichloroethylene, and p-chloronitrobenzene can be improved through the combination of NZVI reduction with anaerobic microorganisms [23, 24]. The removal of COD in integrated microbial-Fe^0^ treatment process also increased when compared with a control anaerobic reactor [17]. Thus, it is obviously worthy of enhancement of anaerobic biodegradation by adding iron-based nanoparticles. However, under the natural environment, using NZVI is highly controlled by its unique physical/chemical properties for treating chlorinated organics [25, 26]. For example, the decreasing degradation rate of the chlorinated contaminants in practice process is mainly due to the particle devitalization and aggregation, and the high surface energy leads to the oxidation of iron nanoparticle in the atmosphere [27].

To overcome these problems, surface modification is recommended for synthesizing more stable and efficient NZVI. The surface modifying agents have been reported including carboxymethyl cellulose, chitosan, silica, and polymeric electrolyte membrane [28], which can be coated onto the surface of the nanoparticles to provide electrostatic repulsion and steric or electrosteric stabilization [14]. Compared with the organic coating materials, SiO_2_ has characteristics of water solubility, nontoxicity, and biocompatibility features. Additionally, the SiO_2_ coating shell has an abundance of surface hydroxyl groups, which makes it easy for surface modification and grafting functional groups [29, 30]. Silica-coated core-shell magnetite nanoparticles, that is, Fe_3_O_4_@SiO_2_, have recently been prepared for potential biomedical applications [31]. Besides, amino-functionalized Fe_3_O_4_@SiO_2_ core-shell magnetic nanomaterial has been prepared as a novel adsorbent for aqueous heavy metals removal [30]. Nevertheless, there are only a few researches about the synthesis and application of amino-functionalized silica-nanoscale zerovalent iron technology to improve the antioxidation abilities in the atmosphere and reduce degradation capacity in the actual water treatment process.

The objectives of this study were to develop a novel amino-functionalized silica-coated nanoscale zerovalent iron (NZVI@SiO_2_-NH_2_) by using a one-step Stöber method with the surface functionalization of NZVI. Batch microcosm experiments were executed to investigate the degradation efficiency of 2,4,6-TCP in the NZVI@SiO_2_-NH_2_/anaerobic granular sludge system and to assess nanoparticles influence on the anaerobic microbial activity. In addition, the effect of NZVI@SiO_2_-NH_2_ dosage and the role of Fe^2+^ on the removal of 2,4,6-TCP were also evaluated. The obtained results in this study will be useful to better understand the feasibility of using NZVI@SiO_2_-NH_2_-anaerobic granular sludge system for the remediation of industrial wastewater with CPs contamination.

## 2. Materials and Methods

### 2.1. Materials

Chemicals ferrous sulfate heptahydrate (FeSO_4_·7H_2_O), ferric chloride hexahydrate (FeCl_3_·6H_2_O), ferrous chloride tetrahydrate (FeCl_2_·4H_2_O), isopropanol, tetraethyl orthosilicate (TEOS, ≥98.0%), and methyl alcohol (99.93%, HPLC grade) and other chemical reagents were purchased from the Jinke Chemical Reagent Co., Ltd. (Guangzhou, China). 3-Aminopropyl trimethoxysilane (APTMS, ≥97.0%), poly(ethylene glycol) (PEG20000, ≥96.0%), and 2,4,6-trichlorophenol (2,4,6-TCP, 98%) were purchased from Aladdin Chemistry Co., Ltd. (China). All chemical solutions and reagents used in the experiments were of analytical grade without further purification.

The tested sludge used as the inoculum for batch experiment was taken from an industrial wastewater treatment plant of pulp and paper mill in Guangzhou. The ratio of volatile suspended sludge to total suspended sludge (VSS/TSS) of this sludge was 0.79. In order to avoid the interference of the residual contaminants in the growth environment for the removal experiments, the anaerobic granular sludge was washed with N_2_-sparged deionized water.

### 2.2. Synthesis of NZVI@SiO_2_-NH_2_

Nanoscale zerovalent iron (NZVI) was firstly prepared using conventional liquid-phase reduction by reducing FeSO_4_ with an excess of NaBH_4_ [14, 32]. The coating iron nanoparticle with silica (NZVI@SiO_2_) was prepared based on a Stöber method using silicon alkoxide as a silica source. In brief, three grams of FeSO_4_·7H_2_O was diluted in the 150 mL ultrapure water/isopropanol (1 : 2, v : v) solution and stirred in a three-neck flask at room temperature. Then, 30 mL of freshly prepared NaBH_4_ solution (1 mol/L) was added dropwise under nitrogen protection, resulting in a suspension of iron nanoparticles. Finally, 4 mL of TEOS and 1 mL NaOH solution were added to the above iron nanoparticle solution to generate the NZVI@SiO_2_.

Amino-functionalized silica-nanoscale zerovalent iron (NZVI@SiO_2_-NH_2_) was conducted on-site by APTMS as the modified agent. After the above coating process for 4 h, 0.8 mL of APTMS was then added to the suspension, and the mixture was preserved at room temperature with continuous stirring (150 rmp/min) for 12 h under nitrogen flow (40 mL/min). After the reaction, the fresh NZVI@SiO_2_-NH_2_ nanoparticles were separated and washed several times with deionized water and ethanol to remove associated polysiloxane and dried overnight at 60°C under vacuum.

### 2.3. Batch Experiments for 2,4,6-TCP Removal

#### 2.3.1. Removal of 2,4,6-TCP by Iron Nanoparticles in Aqueous System

The removal experiments of 2,4,6-TCP by NZVI, NZVI@SiO_2_, and NZVI@SiO_2_-NH_2_ were conducted in 250 mL saline bottles. 2,4,6-TCP (50 mg/L) was firstly spiked into the N_2_-sparged deionized water, and then iron samples (0.5 g/L) including NZVI, NZVI@SiO_2_, and NZVI@SiO_2_-NH_2_ were added to these TCP solutions, respectively. The reaction solution was deoxygenated by N_2_ stream for another 15 min and kept sealed with a rubber stopper during the reaction. The final volume of reaction solution was 200 mL at pH value of 7.0 which was adjusted by HCl or NaOH. The serum bottles were performed in a thermostatic incubator at 35°C with a rotation speed of 150 rpm. At given time intervals, an aliquot of reaction solution was sampled for the measurement of residual 2,4,6-TCP concentration and pH value.

#### 2.3.2. Removal of 2,4,6-TCP by the Integrated NZVI@SiO_2_-NH_2_-Anaerobic Microorganism System

Three parallel nanobiodegradation hybrid systems for 2,4,6-TCP removal were performed by using 250 mL saline bottles with rubber stoppers as anaerobic microcosm reactors: the combination of anaerobic granular sludge system/NZVI  (AGS + NZVI), the combination of anaerobic granular sludge/NZVI@SiO_2_ system (AGS + NZVI@SiO_2_), and the combination of anaerobic granular sludge/NZVI@SiO_2_-NH_2_ system (AGS + NZVI@SiO_2_-NH_2_). The synthetic nutrient solution in each system was deoxygenated at pH 7.0 including a certain amount of 2,4,6-TCP, 10 g VSS anaerobic granular sludge, and the same dose of nanoparticles of 0.5 L^−1^. Two control experiments were also conducted to clarify the intimal activity of anaerobic microorganism and the toxicity of TCP on the biodegradation: a single anaerobic granular sludge system (AGS) was the same as the mixed system, but without any iron nanoparticle; another common anaerobic biodegradation system (AGS only) was the same as AGS, except without the 2,4,6-TCP. The composition of synthetic nutrient solution in biodegradation experiment included 3000 mg/L glucose, 286 mg/L ammonium chloride, and 65 mg/L monopotassium phosphate as nutrient and energy source to facilitate growth of the biomass, with C/N/P ratio of 200 : 5 : 1. In addition, 1 mL stock solution liquor of necessary trace elements was added to the above solution containing the following composition (mg/L): CaCl_2_·2H_2_O, 330; EDTA, 5000; NiCl_2_·6H2O, 190; H_3_BO_4_, 14; ZnCl_2_, 205; MnSO_4_, 500; CuSO_4_·5H_2_O, 250; CoCl_2_·6H_2_O, 240; MnCl_2_·4H_2_O, 205; (NH4)_6_MoO_4_·4H_2_O, 9. The saline bottles were maintained in a constant temperature shaker at 35°C with a rotation speed of 150 rpm. At various time intervals, 2 mL of suspensions was withdrawn from each reactor and control bottle for the analysis of degradation rate of 2,4,6-TCP. Besides, the removal rate, methanogenic activity, and electron transport system (ETS) activity were measured in duplicate.

### 2.4. Analytical Methods

The prepared iron nanoparticles were characterized as follows. The surface morphology and size distribution were determined with an S-3700N scanning electron microscopy (SEM) characterization (Hitachi, Japan). The elemental composition was performed by energy-dispersive spectrometry (EDS, Bruker Quantax, Germany), with energy resolution 123 eV. The crystallographic structures of these nanoparticles and oxides were determined by a D8 Advance X-ray Diffraction system and Bruker AXS with a Cu target and K*α* radiation (*λ* = 0.15418 nm) at 40 kV and 40 mA at 20°C. The scan rate was set at 1.2°/min, and the range was set from 10 to 80 (2*θ*). FT-IR spectra were measured by a Spectrum One-B FT-IR spectrophotometer (Nicolet Magna 550) under dry air at 20°C by a KBr pellets method. Each sample was scanned from 4000 to 400 cm^−1^ with a resolution of 4 cm^−1^. The sampled 2,4,6-TCP reaction solution was firstly centrifuged for 10 min at 10000 g, and the supernatant was filtered through a 0.45 *μ*m hydrophilic polyethersulfone (PES) syringe filter for high-performance liquid chromatography (HPLC) analysis. 2,4,6-TCP was analyzed on a Shimadzu LC-2010A HPLC equipped with a UV detector. A C18 column (250 × 4.6 mm, 15 *μ*m) was used for the separation. The column temperature was set at 30°C. Methyl alcohol and ultrapure water (80 : 20, v/v) were used as the mobile phase. The flow rate was set at 0.7 mL/min. The UV wavelength for 2,4,6-TCP detection was 290 nm. Electron transport system (ETS) activity was measured by an INT method for assessing metal influence on anaerobic sludge [33, 34]. A Shi's fermentation tube was used to measure biogas production. The gas composition of biogas was investigated using gas chromatography (A90, Echrom, China). Methane gas was analyzed with a 2 m × 8 mm stainless column packed with Porapak T (80/100 mesh) and the operational temperatures of detector, injection port, and column were set at 250°C, 200°C, and 100°C, respectively. Argon was used as a carrier gas with a flow rate of 30 mL/min.

## 3. Results and Discussion

### 3.1. Characterization of NZVI@SiO_2_-NH_2_

#### 3.1.1. The Morphology and Composition

**Figure 1:**
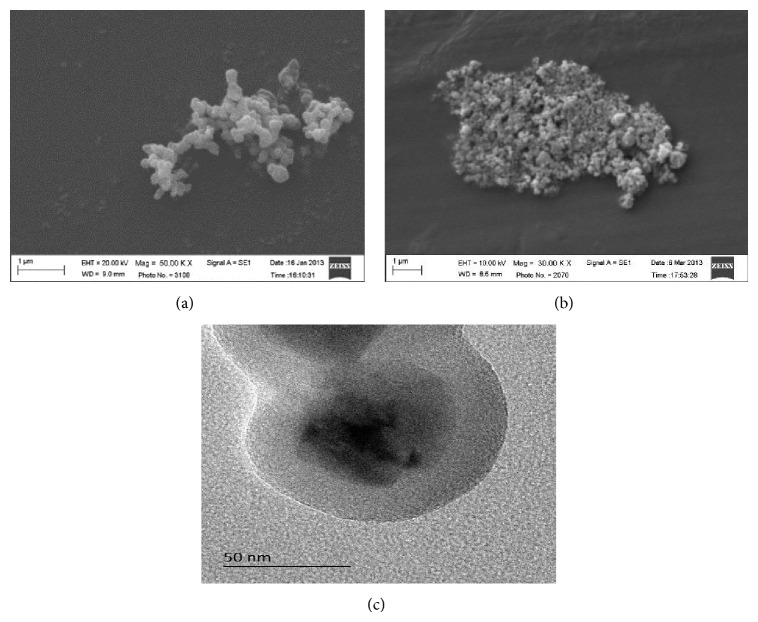
The surface morphology of freshly prepared NZVI@SiO_2_-NH_2_ and NZVI: (a) SEM image of the NZVI@SiO_2_-NH_2_, (b) SEM image of the NZVI, and (c) TEM image of the NZVI@SiO_2_-NH_2_.

The morphology, nanoparticle distribution, and element content of freshly prepared NZVI and NZVI@SiO_2_-NH_2_ were presented in Figure 1. All these freshly synthesized NZVI particles were tightly touching each other in roughly spherical forms (Figure 1(b)), possibly due to their aggregation properties in the aqueous solution and their tendency to remain in the most thermodynamically favorable state. On the contrary, the obtained NZVI@SiO_2_-NH_2_ nanoparticles showed a smoothly spherical shape (Figure 1(a)) and were uniformly covered by an amorphous outer layer of SiO_2_ (Figure 1(c)). SEM image of NZVI@SiO_2_-NH_2_ indicated an average diameter range of about 80–100 nm, while the NZVI particles ranged from 30 to 60 nm. The diameter of iron nanoparticles increased with the coating and surface modification.

**Figure 2:**
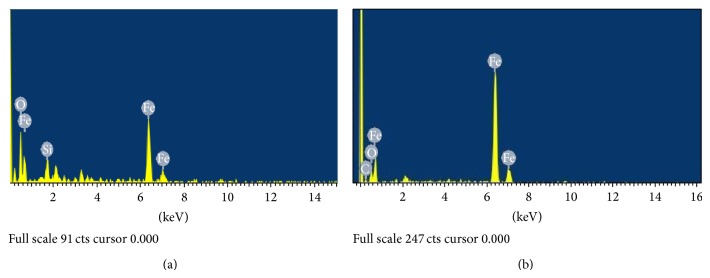
The surface elemental composition of freshly prepared NZVI@SiO_2_-NH_2_ and NZVI: (a) EDS image of the NZVI@SiO_2_-NH_2_ and (b) EDS image of the NZVI.

The chemical composition of NZVI and NZVI@SiO_2_-NH_2_ was also determined by EDS in Figure 2, which can be used to confirm the relation between the changes of particle morphology and the size with the appearing SiO_2_ layer. The chemical composition of NZVI was 0.14 wt% of C, 8.88 wt% of O, and 91.03 wt% of Fe, while the NZVI@SiO_2_-NH_2_ was of 5.9 wt% of Si, 19.45 wt% of O, and 74.65 wt% of Fe, reflecting the coating of SiO_2_ on the NZVI surface. Moreover, the successful amino-functionalization of SiO_2_ layer was also indicated by CHN elemental analysis. The nitrogen content was in a proper range from 0.6 to 0.9% wt. on the NZVI@SiO_2_-NH_2_, while it was not detected on the surface of NZVI. The result confirmed the structure of a SiO_2_ shell on the nFe^0^-core surface and the aminopropyl modification of the SiO_2_ shell.

#### 3.1.2. X-Ray Powder Diffraction and FT-IR Spectroscopy

**Figure 3:**
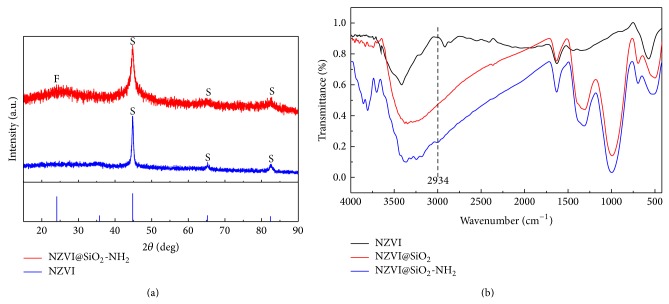
XRD and FT-IR patterns produced the different style iron nanoparticle.

To better understand the primary characterization of the chemical and physical properties of the modified iron nanoparticle, XRD and FT-IR measurements were also made on NZVI and NZVI@SiO_2_-NH_2_ in Figure 3(a). Evidently, the same characteristic peaks were observed for NZVI@SiO_2_-NH_2_ and NZVI with a strong peak at 2*θ* = 44.76°and two weak peaks at 2*θ* = 65.16°and 82.48°, indicative of the body-centered cubic *α*-Fe^0^ in the internals of the modified iron nanoparticle. The crystalline phase of iron nanoparticles was stable during silica coating and surface amino-functionalization process [35]. In contrast to the other characteristic peaks of the NZVI, the NZVI@SiO_2_-NH_2_ showed an extra-large shoulder centered around 2*θ* = 23°, which can be ascribed to the existence of amorphous silica component; and no obvious peaks have shown the existence of the iron oxide as *α*-Fe_2_O_3_ at 2*θ* = 36°. Thus, it proved the presence of the silica shells on the surface of nFe^0^-core after the modified process. The FT-IR spectra for SiO_2_ coating layer and NH_2_-functional group were obviously discerned on the surface of NZVI@SiO_2_-NH_2_ in Figure 3(b). Compared to NZVI, the characteristic peaks of 1057 cm^−1^ in NZVI@SiO_2_ and NZVI@SiO_2_-NH_2_ were attributed to the Si–O–Si asymmetric stretching vibration which indicated the formation of silica shells on the surface of nFe^0^-core. In addition, the typical peak at 2934 cm^−1^ corresponding to –CH_2_– group of aminopropyl from APTMS molecules was attributed to the characteristic peaks of the amine groups, indicating the success of the aminopropyl functionalization onto the surface of NZVI@SiO_2_-NH_2_ particles during the sequential sol-gel process [30].

### 3.2. TCP Dechlorination by the NZVI@SiO_2_-NH_2_ in Aqueous System

**Figure 4:**
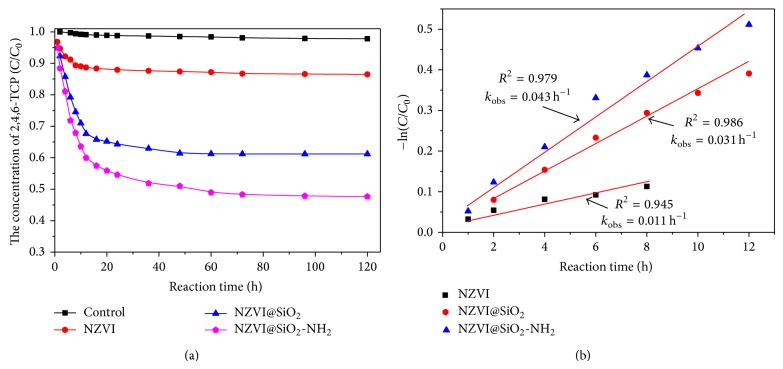
(a) The degradation of 2,4,6-TCP under different iron nanoparticle systems; (b) reaction kinetic plots for the degradation of 2,4,6-TCP versus time.

The reaction reactivity of the iron nanoparticles on 2,4,6-TCP dechlorination could depend on the physical-chemical characteristic of nanoparticles. The effect of modified process on reaction reactivity was evaluated by the degradation efficiency of 2,4,6-TCP in aqueous phase. The results of removal of 2,4,6-TCP by NZVI, NZVI@SiO_2_, and NZVI@SiO_2_-NH_2_ were displayed in Figure 4(b). In the control experiment, the concentrations of 2,4,6-TCP existed steadily over 120 h in anoxic aqueous solution without any iron nanoparticles. The concentration of 2,4,6-TCP decreased obviously with the increasing reaction time by different styles of iron nanoparticles in anoxic conditions. However, for NZVI, only about 14% of the 2,4,6-TCP was degraded within 120 h. On the contrary, NZVI@SiO_2_ and NZVI@SiO_2_-NH_2_ exhibited high reactivity toward 2,4,6-TCP degradation, with the removal rates of 38.9% and 52.3% within 120 h, respectively. On the one hand, the reduction capacity of iron nanoparticles was very sensitive to the water/oxygen in the surrounding media [36]. The freshly prepared NZVI was rapidly corroded and formed an oxidation film, resulting in the loss of reactivity, which may decline and delay the reactivity of further reaction with 2,4,6-TCP. Meanwhile, the higher removal rates of 2,4,6-TCP by NZVI@SiO_2_-NH_2_ and NZVI@SiO_2_ may come from the increased antioxidant capacity by SiO_2_ coating [37]. On the other hand, SiO_2_ cladding layer and NH_2_-functional groups of the modified iron nanoparticles could be used to enlarge the specific surface area and kept more surface active points, leading to the higher surface reactivity and faster contaminants removal rate. The specific surface area analysis showed that NZVI@SiO_2_-NH_2_ and NZVI@SiO_2_ had higher BET surface areas of 126.3 m^2^/g and 107.4 m^2^/g than that of NZVI (67.3 m^2^/g). Besides, the hydrophobic surface properties of NZVI@SiO_2_-NH_2_ were also improved by the introduced functional groups, resulting in good dispersibility, which could effectively improve the chance to contact with the active sites in surface of the nanoparticles and contaminants, thereby increasing the removal efficiency. At the initial reaction, removal of 2,4,6-TCP by NZVI, NZVI@SiO_2_, and NZVI@SiO_2_-NH_2_ under different reaction time could be described by first-order rate equation in different reaction time. The result was presented in Figure 4(b). The obtained *k*_obs_ value was only 0.011 h^−1^ for the NZVI reaction with 2,4,6-TCP. The degradation efficiencies of modified NZVI@SiO_2_ and NZVI@SiO_2_-NH_2_ were 0.031 h^−1^ and 0.043 h^−1^, much higher than that of NZVI, indicating that introducing SiO_2_ cladding layer and NH_2_-functional groups could enhance their ability of reductive dechlorination.

### 3.3. TCP Dechlorination by the Combination of Anaerobic Granule Sludge/NZVI@SiO_2_-NH_2_ System

#### 3.3.1. Influence of NZVI@SiO_2_-NH_2_ on the Anaerobic Biodegradation of 2,4,6-TCP

**Figure 5:**
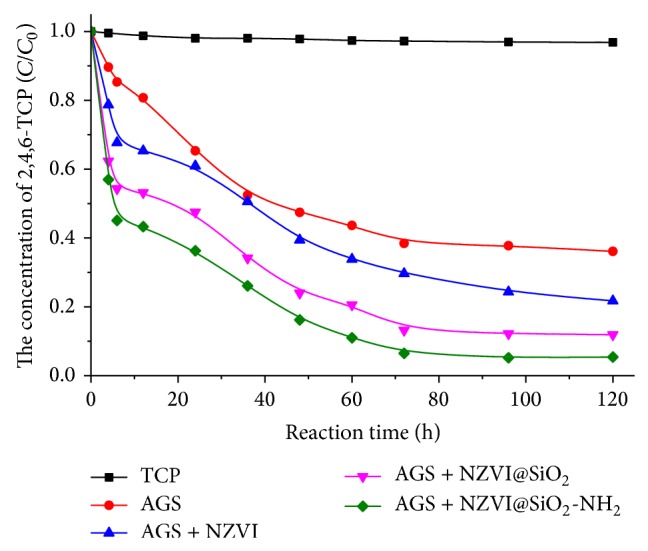
The degradation of 2,4,6-TCP in anaerobic granule sludge system adding different iron nanoparticle.

Application of anaerobic microbial processes for the treatment of chlorinated organic compounds had drawn considerable attention in recent decade [38, 39]. Under unfavorable environmental conditions, maintaining a stable microbial activity during degradation of toxic organic pollutants was one of the challenges in anaerobic treatment process which could lead to irreversible reactor failure. In this study, batch microcosm experiments were used to investigate the microbial activity profiles of the function of NZVI@SiO_2_-NH_2_ on anaerobic granular sludge treating CPs. The influences of NZVI, NZVI@SiO_2_, and NZVI@SiO_2_-NH_2_ on the removal of 2,4,6-TCP in the anaerobic biochlorination system were compared in Figure 5. For the AGS system, anaerobic microorganism alone could biologically degrade 2,4,6-TCP slowly with the removal rate less than 70% during the 120 h experimental period. It was difficult to achieve complete degradation by only microbial action [40]. When NZVI@SiO_2_-NH_2_ was added to anaerobic microbial system, more than 90% of 2,4,6-TCP was removed from the system in 120 h. Consistent with the earlier studies, adding NZVI in anaerobic system was a promising approach to promote anaerobic microbial biochlorination [13]. It is noteworthy that adding the surface-modified nanoparticle (NZVI@SiO_2_-NH_2_ and NZVI@SiO_2_) to anaerobic granule sludge system was also capable of providing positive effect to promote anaerobic biochlorination processes. At the same time, the degradation efficiency of anaerobic microbial system for adding NZVI@SiO_2_-NH_2_, 94.6% of 2,4,6-TCP removal, was substantially higher than that from adding NZVI@SiO_2_ and NZVI, 88.1% and 78.3% of 2,4,6-TCP removal, respectively. The experimental results showed that surface-modified and amino-functionalized NZVI@SiO_2_-NH_2_ could effectively maintain more particle surface activity and improve the dispersibility, thereby improving the ability of the combination system to remove 2,4,6-TCP.

**Figure 6:**
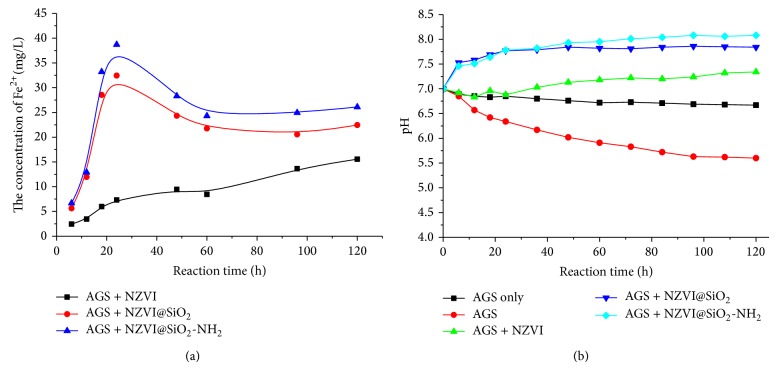
The variation of pH and Fe^2+^ during reaction: (a) the concentration of Fe^2+^ and total iron and (b) pH.

In order to explore the effect of NZVI@SiO_2_-NH_2_ further in anaerobic granule sludge system, the concentration of Fe^2+^ and pH were analyzed. Free ferrous iron were stared as the important iron reagents in practical application of environment. It can be found in Figure 6(a) that the concentration of Fe^2+^ was very low in NZVI/AGS system. In contrast, the concentration of Fe^2+^ went up sharply from 0 to 38.7 mg/L and 32.4 mg/L in NZVI@SiO_2_-NH_2_/AGS and NZVI@SiO_2_/AGS system, respectively. The concentration of Fe^2+^ was released from the Fe^0^ reaction, which could reflect the activity of the iron nanoparticle. However, if the freshly prepared NZVI was exposed in the atmosphere, air and water would trigger a rapid reaction with a large amount of iron corrosion product which formed the oxide layer on the surface of NZVI, identified by the earlier reports as magnetite, maghemite, and lepidocrocite [41]. Once the surface of NZVI was coated by corrosion product, only negligible Fe^0^ reduction reaction would occur, leading to little of Fe^2+^ being released into the reaction medium. Thus, the aminopropyl modification of SiO_2_ shell could effectively slow down the surface passivation of nanoparticles, and ferrous ion could migrate almost freely in the functionalization of layer.

Meanwhile, it has been widely reported that pH was one of the significance factors for the growth of anaerobic microbial activity and the degradation efficiency of chlorinated organic pollutants in anaerobic microbial system. As shown in Figure 6(b), the pH of AGS decreased to 5.6 when adding 2,4,6-TCP to the reaction medium, while the AGS only system was stable at 6.8. This may be due to the inhibition of anaerobic microbial growth and methanogens activity from the toxic chlorophenols, which would produce the accumulation of organic acids. With NZVI@SiO_2_-NH_2_ added, a gradual pH rise was observed in the AGS/NZVI@SiO_2_-NH_2_ system, which could be explained by the fact that H^+^ was required for the dissolution of iron and the iron hydroxide was positively charged by adsorbing H^+^ [42]. Adding NZVI@SiO_2_-NH_2_ could stabilize the reaction environment and maintain the activity of microorganism in the nFe^0^-microbial system. Considering that the reaction medium was unbuffered when the anaerobic granule sludge was exposed to 2,4,6-TCP, the relative change of pH in the nanobiosystem was directly affected by the reaction activity of nanoparticle: the NZVI@SiO_2_-NH_2_ induced increase in pH and was in excess of 0.21 and 0.74 pH units compared to NZVI@SiO_2_ and NZVI at the 120 h of experiment process. Thus, these results proved that NZVI@SiO_2_-NH_2_ had a higher activity than NZVI and NZVI@SiO_2_. Consequently, it is confirmed that the NZVI@SiO_2_-NH_2_ would contribute to the higher removal rate of 2,4,6-TCP, the plenty of electron donors, and the stable environment in the anaerobic granule, which further augmented the function of NZVI in the removal of chlorinated organic compounds.

#### 3.3.2. Influence of NZVI@SiO_2_-NH_2_ on the Anaerobic Microbial Activity

**Figure 7:**
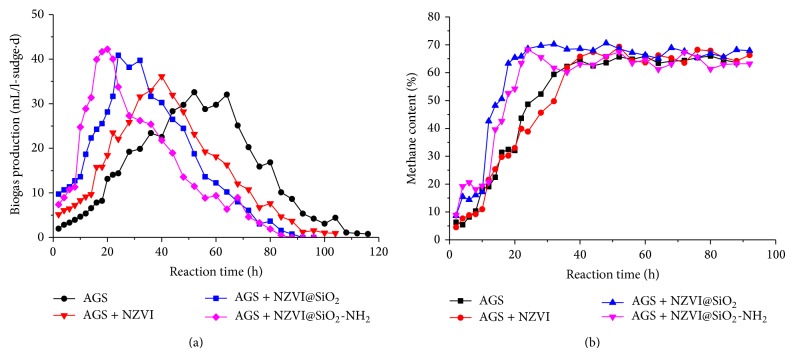
Effect of NZVI@SiO_2_-NH_2_ on the yield (a) and concentration (b) of methane production in anaerobic granule sludge system.

To estimate the actual effect of NZVI@SiO_2_-NH_2_ particles on methane production, the yield of biogas and the content of methane on the combination of AGS/NZVI@SiO_2_-NH_2_, AGS/NZVI@SiO_2_, and AGS/NZVI system and the AGS system were shown in Figure 7 during the operation. The accumulative production of biogas was 273.5 mL, 242.7 mL, and 204.6 mL in the anaerobic system enhanced by NZVI@SiO_2_-NH_2_, NZVI@SiO_2_, and NZVI, respectively, in contrast with 168.3 mL in the control system. In all cases, the yield of biogas was the highest in AGS/NZVI@SiO_2_-NH_2_ system, increasing 12.5% and 33.7% when NZVI@SiO_2_ and NZVI were supplied, respectively. Apparently, the higher activity of NZVI@SiO_2_-NH_2_ available led to plenty of electron donors and a stale and low toxic environment in anaerobic dechlorination process, which could stimulate methanogenesis dramatically. Moreover, the subdued period of methane production was shortened in the anaerobic system enhanced by NZVI@SiO_2_-NH_2_ than AGS/NZVI@SiO_2_, AGS/NZVI, and the AGS system. The highest methane production increased gradually to a daily maximum of 42.2 mL/g VSS d on the 20th hour during the experiment, with methane content increasing 65.4%. Therefore, the present study provided a clear demonstration that the AGS-NZVI@SiO_2_-NH_2_ exhibits better performance in terms of higher methane production and shorter subdued period.

**Figure 8:**
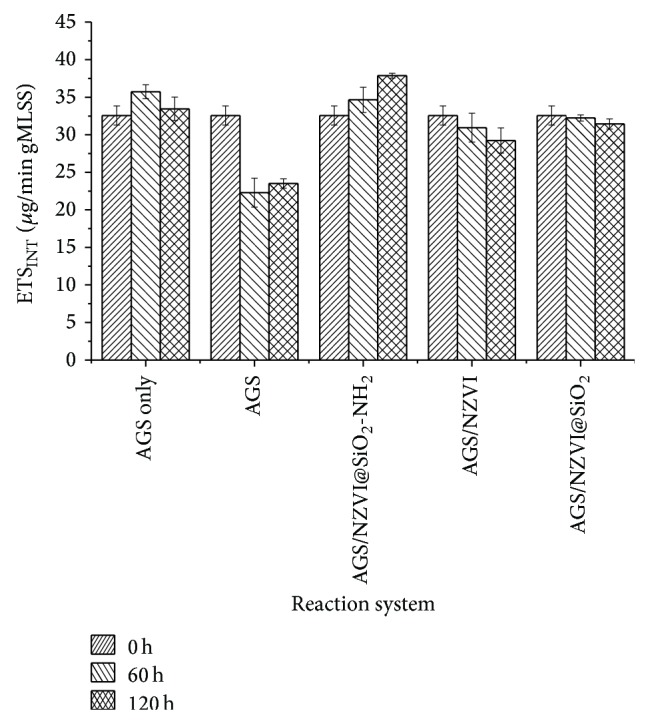
The changes of ETS_INT_ of the anaerobic granule sludge under different concentrations of NZVI@SiO_2_-NH_2_ before and after being exposed to 2,4,6-TCP.

Electron transport system (ETS) activity of the combined AGS/NZVI@SiO_2_-NH_2_ system was also further analyzed by the INT method to describe the influence of NZVI@SiO_2_-NH_2_ on dehydrogenase activity of anaerobic sludge, as shown in Figure 8. The experimental results illustrated that long-term exposure on the 2,4,6-TCP could decrease the activity of anaerobic granule sludge. For example, the ETS activity of the exposed anaerobic granular sludge was 29.7% lower than that of the control granular sludge at all-time points. Meanwhile, the ETS activity of anaerobic granular sludge was stabled at 87.4%, 94.0%, and 113.2% of the control granular sludge after 120 h of experiment process in the combination of AGS/NZVI, AGS/NZVI@SiO_2_, and AGS/NZVI@SiO_2_-NH_2_ system, respectively. NZVI@SiO_2_-NH_2_ did not exert much stimulation and inactivation on the ETS activity of the anaerobic bacteria at the initial process of the experiment, because the core nFe^0^ was avoided directly contacting with microorganism by the surface-modified SiO_2_ shell. It can be observed that the addition of NZVI@SiO_2_-NH_2_ to anaerobic biodegradation system could enhance the activity of sludge and reduce the adverse influence of 2,4,6-TCP.

### 3.4. Effect of NZVI@SiO_2_-NH_2_ Dosage

**Figure 9:**
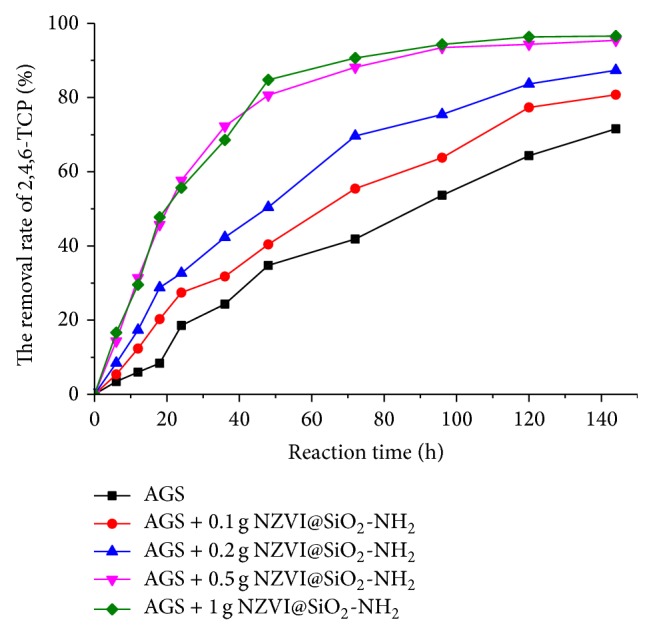
The degradation curves of 2,4,6-TCP by the anaerobic treatment process (*T* = 35 ± 0.1°C, pH = 7.0, initial concentration of 2,4,6-TCP (*C*_0_) = 50 mg/L, anaerobic granular sludge = 10 g VSS, and stirring rate = 150 rpm).

Four identical batch microcosm experiments were operated at different dosages of 0.1, 0.2, 0.5, and 1 g/L in parallel for 120 hours to evaluate the removal rate of 2,4,6-TCP. The removal rate of 2,4,6-TCP as a function of time was presented in Figure 9. It can be observed that high removal rate of 2,4,6-TCP occurred when the NZVI@SiO_2_-NH_2_ dosage increased in the anaerobic granular sludge system. For example, the removal rate of 2,4,6-TCP was found to be 80.7%, 87.4%, 95.4%, and 96.6%, respectively, when the addition dosage of NZVI@SiO_2_-NH_2_ to the anaerobic system was 0.1, 0.2, 0.5, and 1 g/L. In addition, the concentration of 2,4,6-TCP in combined system was obviously deceased in the first 10–12 h of the experiment, which indicated that 2,4,6-TCP can be directly chemically reduced and/or be adsorbed on the surface layer of NZVI@SiO_2_-NH_2_. The adsorptive and active sites on the surface of NZVI@SiO_2_-NH_2_ increased when the amount of NZVI@SiO_2_-NH_2_ increased. Moreover, adding NZVI@SiO_2_-NH_2_ dosage to anaerobic granule sludge system is capable of providing more electron donors to promote anaerobic metabolic processes, and the corrosion process of NZVI@SiO_2_-NH_2_ can produce Fe^2+^/Fe^3+^ and hydrogen which can be used as minerals for the anaerobic microorganisms; thereby, the remnants of pollutants and toxic intermediate products could be further removed biologically by the attached microorganism in the combined system.

**Figure 10:**
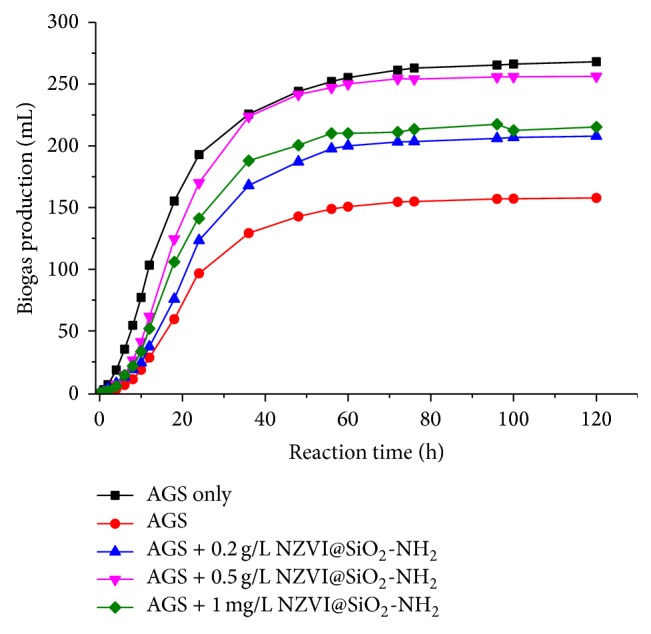
The production curves of CH_4_ in different anaerobic treatment process with and without NZVI@SiO_2_-NH_2_ at pH 7.0.

Methanogenic activity test was used to determine the influence of NZVI@SiO_2_-NH_2_ dosage on the anaerobic sludge activity in various anaerobic processes.  Figure 10 showed the summary of the cumulative biogas productions with the function of NZVI@SiO_2_-NH_2_ in anaerobic methanogenic process. The accumulative production of biogas of the seed granular sludge at 35°C was 268.1 mL in the AGS only system. When the anaerobic system was exposed to 50 mg/L of 2,4,6-TCP, the cumulative production of biogas decreased to 157.7 mL, far lower than the AGS only. The reason may be that chemical 2,4,6-TCP had a significant inhibitory effect on methanogenic activity of anaerobic microorganism, which affected the 2,4,6-TCP biological dechlorination and final mineralization by anaerobic microorganism. Maintaining a sufficient microbial activity was essential for a stable anaerobic treatment system on unfavorite condition. With increasing the dosage of NZVI@SiO_2_-NH_2_ from 0.1 g/L, 0.2 g/L, and 0.51 g/L to 1 g/L, obvious increase of the cumulative production of biogas in the integrated AGS/NZVI@SiO_2_-NH_2_ system during the operation was observed. And among these dosages of NZVI@SiO_2_-NH_2_, the cumulative production of biogas (0.2 g/L and 0.5 g/L) increased by 31.7% and 62.4%, respectively, more than that of the AGS system at 120 h. That is to say, the methanogenic activity of the seed sludge increased with the increasing dosage of NZVI@SiO_2_-NH_2_. The results implied that the activity of anaerobic microorganisms was significantly influenced by the function of the added iron nanoparticles. However, the results of cumulative biogas productions in anaerobic dechlorination processes with the different dosage of NZVI@SiO_2_-NH_2_ were largely different from the removal rate of 2,4,6-TCP (Figure 9). The methanogenic activity of 1 g/L of NZVI@SiO_2_-NH_2_ was considerably lower than that of 0.5 g/L, which means that the anaerobic system should be operated with appropriate concentration of iron nanoparticles.

### 3.5. Effect of the Initial Concentration of Fe^2+^

**Figure 11:**
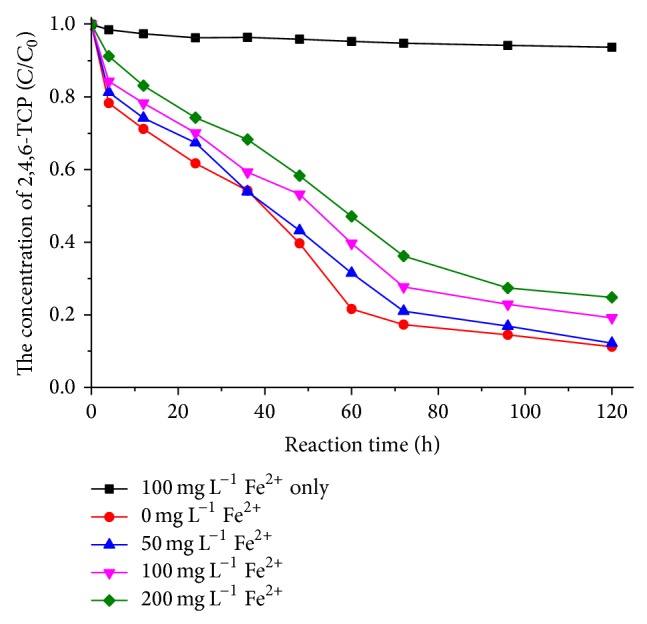
The effect of Fe^2+^ on the removal of 2,4,6-TCP in NZVI@SiO_2_-NH_2_-anaerobic granule sludge system.

The effect of Fe^2+^ on the removal of 2,4,6-TCP in combined anaerobic granule sludge/NZVI@SiO_2_-NH_2_ system was investigated at initial neutral pH, as shown in Figure 11. When only 100 mg/L of Fe^2+^ was presented in the reaction solution, the concentration of 2,4,6-TCP kept near 48 ± 0.1 mg/L within 120 h. However, the existence of Fe^2+^ may involve the reactions (Fe^2+^ → Fe^3+^ + e^−^) which might be attributed to increasing Fe^2+^ reducing ability. The result obviously indicated that the ferrous ion was not the major source on the removal of 2,4,6-TCP. When 50 mg/L to 200 mg/L Fe^2+^ was added to the AGS/NZVI@SiO_2_-NH_2_ system, the removal rate of 2,4,6-TCP decreased from 87.8% to 75.2%, indicating that the ferrous ion in the mixed system of NZVI@SiO_2_-NH_2_ and anaerobic sludge did not enhance the 2,4,6-TCP degradation. The higher content of Fe^2+^ had a negative effect on the 2,4,6-TCP degradation in the integrated AGS/NZVI@SiO_2_-NH_2_ system. Besides, Table 1 has shown that the added Fe^2+^ had a significant inhibitory effect on methanogenic activity of AGS/NZVI@SiO_2_-NH_2_ system. The specific methanogenic activity decreased from 48.7 mL/g VSS d to 37.5 mL/g VSS d at 200 mg/L of Fe^2+^. This may be because higher concentration of Fe^2+^ (200 mg/L) decreased very quickly in the reaction system with a large amount of amorphous colloidal Fe(OH)_3_. These iron precipitates can be attached to the surface of iron nanoparticles and anaerobic granular sludge, which would decrease the reduction ability of iron nanoparticles and the activity of anaerobic microorganisms. Thus, this would further affect the degradation efficiency and methanogenic activity of anaerobic microorganisms. Besides, the concentration of Fe^2+^ gradually raised and remained at a certain concentration in this combined system (Figure 6(a)), suggesting that the added NZVI@SiO_2_-NH_2_ could continue to release Fe^2+^ as the supply of electronics and elements for NZVI@SiO_2_-NH_2_-anaerobic granule sludge system.

## 4. Conclusion

NZVI@SiO_2_-NH_2_ was successfully synthesized by the surface functionalization of NZVI using TEOS and APTMS. The obtained NZVI@SiO_2_-NH_2_ had a good dispersibility and antioxidant capacity and can be stored in the air for long time. Compared to the NZVI, NZVI@SiO_2_-NH_2_ showed an appreciable reactivity with 2,4,6-TCP. The determined *K*_obs_ was 0.043 h^−1^ at the neutral condition, much higher than that of NZVI. The combined anaerobic granule sludge system/NZVI@SiO_2_-NH_2_ had significant synergistic effects on the removal of 2,4,6-TCP. More than 94.6% of 2,4,6-TCP was removed from the combined AGS/NZVI@SiO_2_-NH_2_ system during the operation processes. The added NZVI@SiO_2_-NH_2_ to microbial system can decrease the toxic inhibition of 2,4,6-TCP, resulting in improved cumulative amount of methane production and ETS activity. Moreover, the combination of AGS/NZVI@SiO_2_-NH_2_ system should be operated with appropriate concentration of NZVI@SiO_2_-NH_2_. The 2,4,6-TCP degradation and methane production with extra Fe^2+^ (>50 mg/L) in the combined AGS/NZVI@SiO_2_-NH_2_ were remarkably adverse on the performance and methanogenic activity of AGS-NZVI@SiO_2_-NH_2_. The novel of modified nanoparticle could be an effective and promising material in the anaerobic treatment system for removal of CPs from industrial wastewater. However, further study should be carried out to control the efficiency and activity of microbial system in application of the novel NZVI@SiO_2_-NH_2_ in situ remediation of industrial wastewater.

## Figures and Tables

**Table 1:** The effect of Fe^2+^ on methanogenic activity of NZVI@SiO_2_-NH_2_/anaerobic granule sludge system.

Fe^2+^ dosage (mg/L)	Maximum gas output (mL/h)	Specific methanogenic activity (mL/g VSS d)	Relative activity
0	15.8 ± 0.62	48.72 ± 2.20	—
50	13.3 ± 0.75	45.67 ± 0.67	93.74%
100	10.5 ± 1.05	42.59 ± 2.25	87.42%
200	9.7 ± 0.43	37.56 ± 0.93	77.09%
